# Cell-Free Gene Expression in Bioprinted Fluidic Networks

**DOI:** 10.1021/acssynbio.4c00187

**Published:** 2024-07-23

**Authors:** Alexandra Bienau, Anna C. Jäkel, Friedrich C. Simmel

**Affiliations:** TU Munich, School of Natural Sciences, Department of Bioscience, 85748 Garching b. München, Germany

**Keywords:** cell-free gene expression, bioprinting, microfluidics, hydrogels

## Abstract

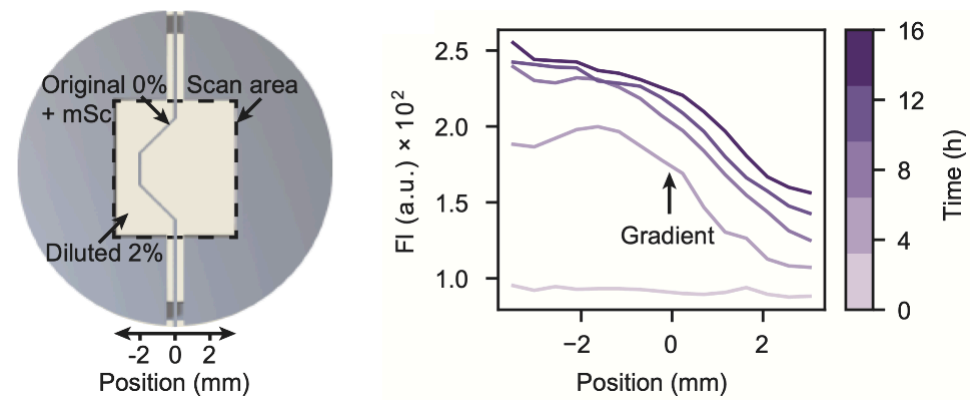

The realization of soft robotic devices with life-like properties requires the engineering of smart, active materials that can respond to environmental cues in similar ways as living cells or organisms. Cell-free expression systems provide an approach for embedding dynamic molecular control into such materials that avoids many of the complexities associated with genuinely living systems. Here, we present a strategy to integrate cell-free protein synthesis within agarose-based hydrogels that can be spatially organized and supplied by a synthetic vasculature. We first utilize an indirect printing approach with a commercial bioprinter and Pluronic F-127 as a fugitive ink to define fluidic channel structures within the hydrogels. We then investigate the impact of the gel matrix on the expression of proteins in *E. coli* cell-extract, which is found to depend on the gel density and the dilution of the expression system. When supplying the vascularized hydrogels with reactants, larger components such as DNA plasmids are confined to the channels or immobilized in the gels while nanoscale reaction components can diffusively spread within the gel. Using a single supply channel, we demonstrate different spatial protein concentration profiles emerging from different cell-free gene circuits comprising production, gene activation, and negative feedback. Variation of the channel design allows the creation of specific concentration profiles such as a long-term stable gradient or the homogeneous supply of a hydrogel with proteins.

## Introduction

In recent years, the scope of robotic systems has significantly expanded to include also molecular, nanoscale, and biological components, which has resulted in unconventional approaches toward robotics that operate across vastly different length scales. For instance, soft robotics has integrated elastic and compliant materials with novel actuation principles to enable applications in healthcare, wearables, handling of fragile objects, and many others. Live cells like cardiomyocytes^1−4^ or other engineered multicellular assemblies (xenobots^5,6^) were utilized for applications in “living” millimeter-scale robotics.^7^ Integrated molecular assemblies, most notably DNA-based nanostructures, have been developed that exhibit basic robotic functions such as sensing, computation, and actuation at the nanoscale.^8−11^ Rapid recent advancements in protein fold prediction and design^12−14^ promise to add ever more versatility and functionality to molecular robotic systems.

We anticipate that in the future the boundaries between robotics, molecular systems engineering and synthetic biology will continue to blur, resulting in hybrid robotic systems, in which biological and abiotic components are intimately connected. Such systems are expected to have advanced (bio)chemical sensing capabilities and the ability to programmably produce (bio)molecules on demand. A promising approach for the execution of such biochemical functions is to utilize cell-free technology,^15^ which has the advantage of not being restricted by the complex requirements of living systems and can be integrated in synthetic materials.^16^ Cell-free gene expression reactions are typically performed in cell extracts that are supplemented with biosynthetic building blocks (NTPs and amino acids) and biochemical cofactors (e.g., ATP, coenzyme A), allowing for high yield in situ production of proteins.^17−20^

A wide variety of proteins have been synthesized in cell-free systems, ranging from transcription factors to fluorescent proteins, enzyme cascades, protein switches, and even whole bacteriophages, with applications found in biosensing, biomanufacturing, biomedicine and bottom-up synthetic biology.^15,20^ Until now cell-free expression systems only have a very limited metabolism and are unable to “replicate” as living cells - only partial regeneration of fuel molecules or other components of the cell extract has been demonstrated.^21−23^

The lack of powerful metabolic activity has a major impact on the capabilities and implementations of cell-free systems. When compartmentalizing cell-free reactions into liposomes or emulsion droplets—a typical approach toward the creation of synthetic cell-mimicking systems—the lifetime of the reactions and thus the complexity of the executable functions is very limited, as the systems quickly run out of energy and degrade. To circumvent these issues, various approaches have been developed to conduct cell-free reactions in open systems that allow material and energy exchange with the environment. For instance, cell-free expression has been performed for extended periods of time using microfluidic reaction chambers that contain surface-immobilized gene brushes.^24−26^ In such a system, fresh cell extract and metabolites can be supplied in continuous flow and thus allow operation for many days. Alternatively, cell-free reactions can be performed in microfluidic chemostats, in which a fraction of the reaction mix, including the coding DNA, can be replenished at regular intervals.^27−30^

Yet another approach uses hydrogels to immobilize DNA for gene expression, which allows supply of reaction mix and nutrients through the porous gel matrix. For instance, DNA-loaded hydrogel particles^31^ have been shown to allow long-lived gene expression^32,33^ and were also used to execute several incompatible reactions in parallel.^34^ Gel particles were also utilized as mimics of cell organelles,^35,36^ and have been envisioned as components for smart hybrid biomaterials capable of differentation and pattern formation at the millimeter scale.^37^

In the present work we sought to merge these approaches using mm-scale gene-expressing gel matrices that are supplied via custom-shaped bioprinted microfluidic vasculature. Our bioprinting approach allows for the rapid and inexpensive implementation of various channel network designs, which helps to explore their impact on the spatiotemporal dynamics of gene expression. For the gel matrix we utilized agarose, which was recently found to be a promising material for embedded cell-free protein synthesis.^38^ Direct printing of hybrid agarose hydrogels containing a cell-free expression system presents significant experimental challenges, however. These result primarily from the substantial volume requirements (several mL) and the high temperatures (up to 65 °C^39^) needed for agarose printing, which lead to elevated material costs and risk the denaturation of sensitive components within the cell extract. Inspired by previous work on perfusable vascularization of cell-laden hydrogels,^40,41^ we therefore adopted an indirect printing approach to create channel structures using a fugitive ink, which is removed after casting the cell extract containing hydrogel on top. Additional cell-free reaction mixture can be injected into the channel to create spatial organization.

In the following, we first demonstrate the technical implementation of our printing approach. We then study how the gel matrix itself affects gene expression in the cell-free system in bulk. Using different cell-free gene circuits and fluidic channel structures, we then show how spatial preorganization by an artificial vasculature can be utilized to control the spatiotemporal dynamics of gel-embedded cell-free expression reactions.

## Results

### Bioprinted Vascularized Hydrogels

**Figure 1:**
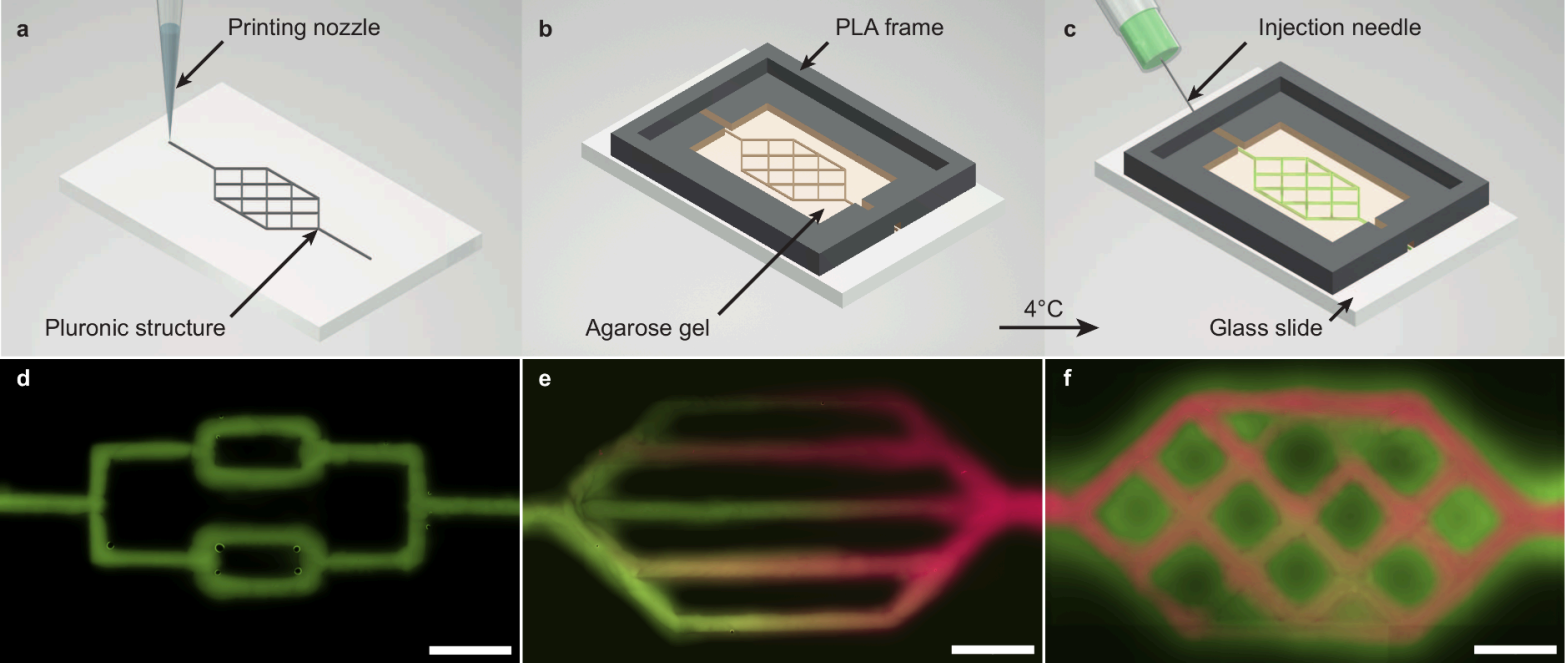
Fabricating vascularized hydrogels. (a–c) Pluronic F-127 is deposited at room temperature onto a glass slide using an extrusion-based bioprinter to create sacrificial channel structures. Subsequently, a PLA frame is adhered to the slide, creating a container to cast preheated agarose onto the structure. The construct is then cooled to 4 °C, resulting in the gelation of agarose and liquefaction of Pluronic F-127. This allows to remove the sacrificial structures so that other solutions can be injected into the hydrogel channels, using a syringe needle. (d–f) We filled different channel structures within agarose hydrogel, each having two inlets, with fluorescent dye solutions for visualization. In (d), fluorescein (green) was injected into a hierarchically branched network with two levels and imaged immediately. (e) Another branched structure with five parallel channels was filled with fluorescein (green) solution from one side and rhodamine (magenta) from the other side so that mixing occurred in the central region. In (f), we filled a grid with fluorescein (green) first, and, after diffusion into the hydrogel for 5 min, injected rhodamine (magenta) second. The scale bars represent 3 mm each.

Custom-designed channel structures within agarose hydrogels were fabricated by indirect printing, using an extrusion-based bioprinter and Pluronic F-127 as a fugitive ink (Figure 1a–c). To evaluate the fabricated structures, fluorescent dye solutions were injected for visualization. Various designs were tested, including straight, curved, and branching components (Figure 1d–f, SI Figure S1), which demonstrated the feasibility of creating reasonably complex structures on a centimeter scale, analogous to natural perfusion networks.

For Figure 1d,e, images were captured immediately after injection, before the dye could diffuse into the surrounding agarose hydrogel, to recapitulate the channel structures. Their widths depend on the line widths of the sacrificial structures, which are constrained by the inner diameter of the printing nozzle, but are also influenced by the printing parameters applied and the structure design. We selected a nozzle with an inner diameter of 0.2 mm and used minimal printing pressures necessary to form consistent lines. The resulting channel widths varied between the different samples and within individual structures, such as between straight segments and branching points. Fine channels narrower than 1 mm were consistently achieved throughout the structures investigated.

The structure in Figure 1f was consecutively injected with two different dyes. After the addition of fluorescein solution, we allowed the dye to diffuse into the gel for 5 min, and then injected a solution containing rhodamine. The resulting image illustrates how a dense vascular network can, in principle, be used to supply small molecules throughout the gel quickly.

### Cell-Free Protein Expression in the Gel Matrix

For the implementation of cell-free protein synthesis within the fabricated channel systems, one of the key challenges is the compatibility of the reaction mixture with the hydrogel. In comparison to traditional PDMS-based microfluidic devices, reaction components are not confined to the channels by impermeable boundaries, but can diffuse into the surrounding hydrogel. Interactions of the reaction mix with the hydrogel itself as well as its dilution by diffusion could thus potentially impair protein synthesis.

**Figure 2:**
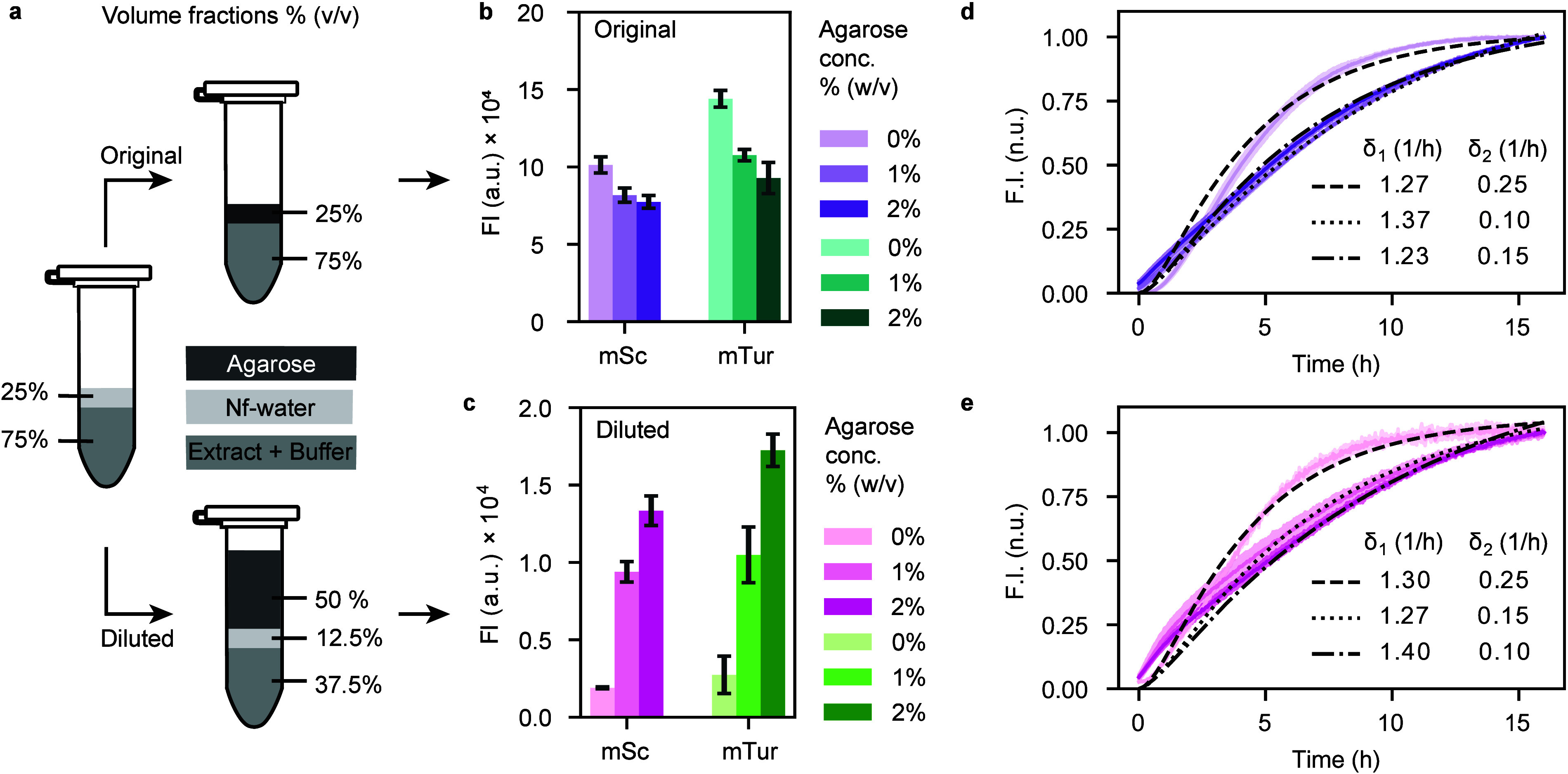
Cell-free protein synthesis in agarose hydrogels. (a) Two methods to incorporate reaction components into agarose hydrogels, based on a standard reaction mixture containing 75% extract and 25% buffer. The original volume fractions were either kept by substituting the nuclease-free (nf) water fraction with concentrated agarose solution or they were decreased by adding twice concentrated agarose in the same volume. This dilution resulted in a mixture with only 37.5% extract and buffer, the final template plasmid concentration was maintained constant at 2 nM in all mixtures. (b) Fluorescence intensity values for cell-free protein synthesis of mScarlet-I (mSc) in purple (left) and mTurquoise2 (mTur) in turquoise (right) after 16 h in original mixtures. The final agarose concentrations 0%, 1%, 2% are indicated by different shadings of the color bars. (c) Fluorescence intensity values of mSc (pink, left) and mTur (green, right) after 16 h produced in diluted mixtures with different agarose concentrations. (d) Kinetic curves of mSc cell-free synthesis in original agarose hydrogel mixtures of different concentrations, normalized by fluorescent intensity end point values. Colored lines with filled areas represent the measured data with standard deviation, and dashed (0%), dotted (1%), and dashdotted (2%) lines indicate the corresponding fit curves with the fit values displayed. (e) Kinetic curves of diluted mixtures, normalized with the end point values. Dashed, dotted, and dashdotted lines represent the fit curves with the given fitting parameters.

We investigated the impact of these effects by creating different mixtures of the cell-free components with agarose (Figure 2 and SI Figure S2). The compositions were based on a standard cell-free reaction containing 33% *E. coli* cell-extract (homemade TXTL following the protocol published in^42^), 42% buffer, and 25% nuclease-free (nf) water to fill up the volume. One set of reaction mixtures - termed original mixtures in the following - was composed of the same ratios, but with agarose solutions of different concentrations replacing the 25% nf water fraction. Original mixtures thus had the same concentration of cell-free components as the initial expression system, but differed in their agarose concentration (Figure 2a, top).

Three samples were created with final agarose concentrations ranging from 0% to 2% and the production of two fluorescent proteins, mScarlet-I (mSc) and mTurquoise2 (mTur), was tested to exclude protein-specific effects. The second reporter, mTur was expressed from a plasmid including a promoter specific for the sigma factor 28 (σ_28_), which required the coexpression of that factor from another plasmid. After performing the reaction for 16 h, fluorescence intensity values of the agarose samples were on the same order of magnitude as the 0% controls, but were slightly decreased in comparison (Figure 2b).

In all samples, the active expression of mTur in the presence of σ_28_ can be clearly distinguished from background synthesis without providing the corresponding plasmid (cf. SI Figure S3). Accordingly, the cell-free reaction showed only minor impairment by the hydrogel matrix, possibly caused by the reduced diffusion of components within the gel. Increasing the agarose concentration from 1% to 2% had a marginal impact on the yield of the reaction.

A second set of mixtures (“diluted mixtures”) was created to investigate the effect of dilution on the cell-free reaction. In this case, the standard reaction mixture was prepared as before, but now followed by mixing with an equal volume of nf water or agarose (Figure 2a, bottom). The final concentrations of the cell-extract and buffer were thus reduced by half. Template plasmids were added such that their final concentrations were identical to those used in the original mixtures. As shown in Figure 2c, after 16 h a measurable protein production was achieved in all samples, however, with a significant drop in performance. In comparison with the original mixtures, the final protein concentrations achieved were reduced by about 1 order of magnitude, which is considerably lower than naively expected from the 2-fold dilution of the extract.

Interestingly, in contrast to our findings with the original mixtures, adding hydrogel to the diluted mixtures significantly enhanced gene expression efficiency. This resulted in a 10-fold increase in expression levels when the agarose concentration was raised from 0% to 1%, and an even greater increase at 2% agarose. The same effect was observed for GFP expression in commercial myTXTL cell extract with an almost 2-fold increase, see SI Figure 2.

Molecular crowding^43^ has been previously observed to have opposing effects on gene expression dynamics that can result in an overall decreased (as seen for the original mixtures) or increased expression level (as for the diluted mixtures), depending on the concentration of the reactants and the crowding level.^44−46^ We surmise that the enhanced expression in diluted mixtures caused by the addition of agarose results from the free volume exclusion by the gel, which leads to an effective up-concentration of the components in the pores of the agarose matrix. By contrast, in the original solution, hindered diffusion appears to be the dominant effect.

As shown in Figure 2d,e, the presence of the gel had a major impact on the expression kinetics for both the original and diluted samples. Interestingly, however, when comparing original and diluted samples of the same gel concentration, the kinetic behavior appears to be very similar, which we highlighted in the Figure by normalizing the curves to their end point fluorescence values.

To quantitatively describe the differences between the kinetic curves, we fitted a simplified model of the cell-free expression process to the data. The shape of the kinetic curves shows that the final product (the fluorescent protein) is stable, indicating that protein degradation is negligible on the time scale of the experiment. Furthermore, the sigmoidal shape of the 0% agarose curves suggests that gene expression involves at least two kinetic steps occurring at similar time-scales. The fact that dilution does not appear to affect the curve shape indicates that these steps obey first order kinetics.

We considered several candidate processes for these steps. The expression of a fluorescent protein requires transcription of mRNA, translation, and maturation of the fluorescent protein. In addition, the cell-free expression system is known to lose activity over a time course of a couple of hours. As the mRNA lifetime has been previously reported to be on the order of only 10 min in cell extract,^47^ we assume that mRNA levels quickly reach steady state concentrations. We therefore focused on translation, protein maturation and degradation of the cell extract as the dominant processes, resulting in the following relation for the concentration of the fluorescent protein as a function of time:
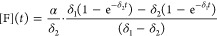
1In this model, the two dominant time-scales τ_1_ = 1/δ_1_ and τ_2_ = 1/δ_2_ correspond to the time required for expression and maturation of the protein and the lifetime of the extract, respectively (cf. the SI for details of the model). Empirically, the lifetime τ_2_ of a cell-free expression system is on the order of a few hours.^21,48^ Rather than fitting both δ_1_, δ_2_ simultaneously, we tested a series of δ_2_ values in the range from 0.05 h^–1^ to 0.2 h^–1^, and determined the δ_1_ by curve fitting of the normalized data for each fixed δ_2_ (SI Figures S4–S6). We identified the best sets of fit curves for each sample (central plot each), which are shown in Figure 2d,e. The model fits suggest that the value for δ_1_ lies in the narrow range ≈1.23–1.4 h^–1^ for all samples and thus corresponds well with a rate expected for the maturation of a fluorescent protein (τ_1_ ≈ 40–50 min).^49^ As discussed in the Supporting Information, δ_1_ might have to be taken as an effective rate constant, however, as there is no clear time scale separation between RNA and protein expression kinetics. Notably, the resource degradation rate δ_2_ appears to be smaller within the hydrogel for both the original and diluted samples. Whereas gene expression appears to be active for ≈4 h in the samples without gel, the lifetime of the reaction is extended to ≈7–10 h in the hydrogel samples. As the protein yield of the diluted mixture was observed higher with agarose than without, this behavior cannot be attributed to higher resource consumption.

### Spatiotemporal Expression Dynamics in Hydrogels

For experiments in our channel systems, we chose to use the standard mixture with nf water in the channels, enabling fast production and high final protein concentrations. As dilution drastically diminished protein synthesis yield, supplying cell-free reaction mixture not only in the channels but also through the surrounding gel was crucial to prevent the rapid loss of cell-free components by diffusion along the concentration gradient into the hydrogel. To reduce consumption of the valuable cell-extract in the relatively large gel areas, we opted for a diluted 2% mixture, which ensured a stable gel matrix, moderated the concentration gradient, and was found to maintain a reasonable level of protein production.

**Figure 3:**
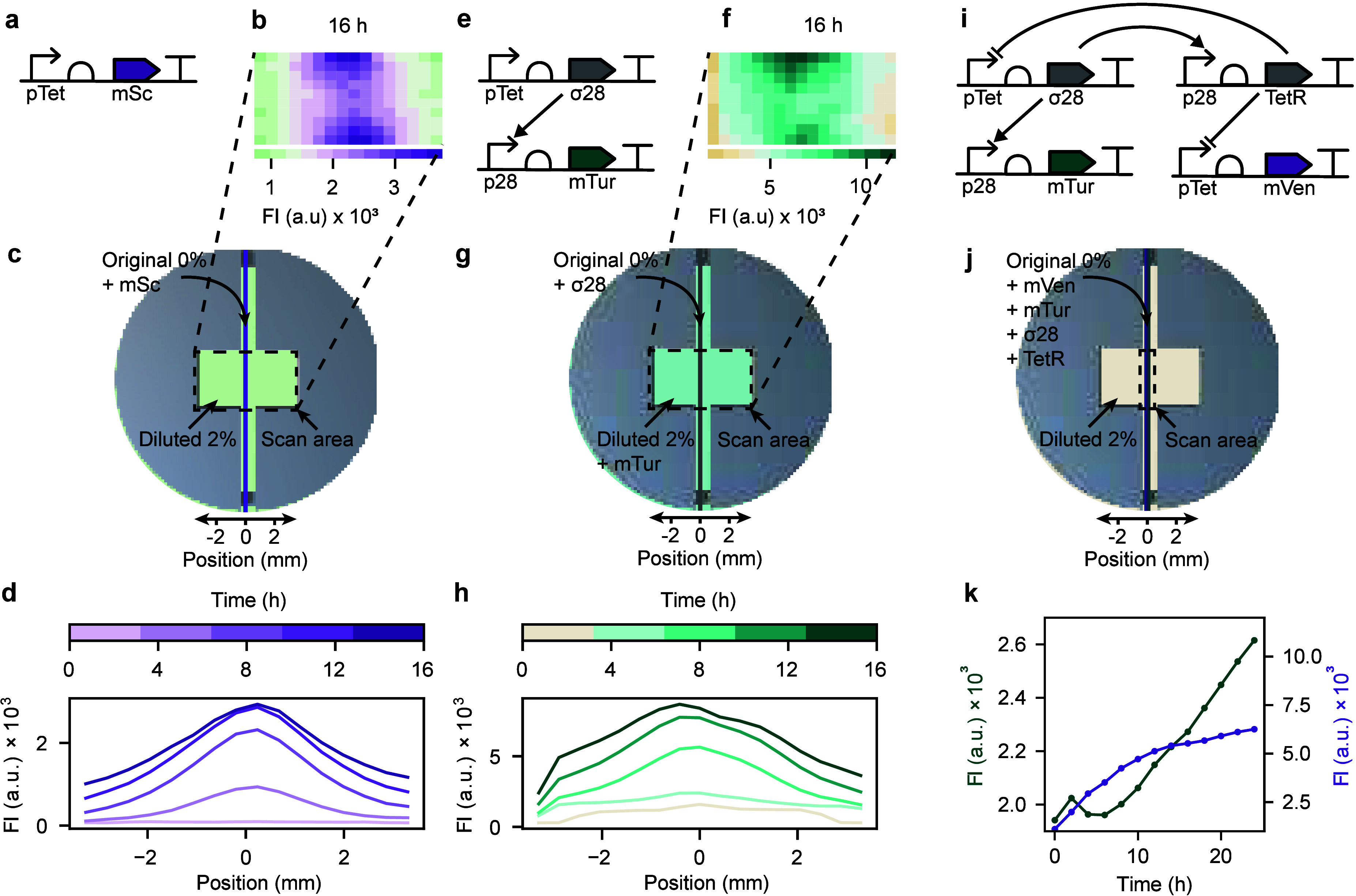
Cell-free protein synthesis and diffusion in single-channel hydrogels. All samples were constructed as shown in the schematics (c, g, j), featuring a central channel within an agarose hydrogel, created using a 0.6 mm syringe needle mold. The structures were fabricated as rectangular shapes of size 7 × 4 mm^2^ within circular PLA containers to fit multiwell plates for measurement (cf. SI Figure S8). Channels in the gel were filled with original mixtures containing 75% cell-free extract and buffer plus different plasmids. The hydrogels were composed of the diluted mixture with 2% agarose and 37.5% extract and buffer. (a–d) Production of mScarlet-I (mSc) in a central channel. The template plasmid shown in (a) was supplied into the channel. The increase in fluorescene intensity by mSc production is represented in a heat map after 16 h (b) and in profile plots over time (d). (e–h) Induction of mTurquoise2 (mTur) from a central channel. The circuit given in (e) is spatially divided such that the sigma factor 28 (σ_28_) is produced within the channel, while the mTur plasmid is incorporated into the agarose hydrogel. Changes in fluorescence intensity resulting from mTur expression are shown in a heat map (f) of the scan area after 16 h and in temporal profile plots (h). The profile plots in (d, h) were generated by averaging the scanned area values in the vertical direction and plotting them against the horizontal coordinates. (i–k) Feedback gene circuit in a central channel. The circuit shown in (i) comprises two regulatory proteins: σ_28_ as an activator, TetR as a repressor, along with two fluorescent reporter proteins mTur and mVenus (mVen). A negative feedback loop is established by σ_28_ activating expression of TetR, which in return represses inducer expression. The fluorescence intensities of the reporter proteins were measured and averaged over the scanned area depicted in (j), with resulting values plotted over time in (k).

Figure 3 presents three examples for simple “vascularized” systems, in which central supply channels were embedded into rectangular shaped agarose hydrogels with sizes of 7 × 4 mm^2^. These were fabricated by casting agarose around a syringe needle that served as a mold within printed PLA containers to fit into multiwell plates for measurement (SI Figure S7).

In the experiments shown in Figure 3a–d (and SI Figure S9a–c), the central channel was used to supply the system with the mSc template plasmid (Figure 3a) in cell extract (original mixture, 0% agarose), whereas the surrounding 2% agarose gel did not contain any plasmid, but was prepared with diluted cell extract. We assume the plasmids to predominantly remain within the channel region. With a size of *N*_bp_ ≈ 3000 bp the radius of gyration of the coiled plasmids is expected to be , with *L* = *N*_bp_ × 0.34 nm and *L*_p_ = 50 nm the contour and DNA persistence lengths, respectively. Thus, their size is on the order of the ≈200 nm pore size of the 2% agarose hydrogels.^50^ We therefore consider diffusion of the plasmids into the gel negligible on the time scale of our experiment.

We determined the fluorescence level at different locations in the gel using a laser scanning system with a pixel size of (250 μm)^2^ (Figure 3b, Methods). Time-resolved profile plots obtained from these data showed that protein synthesis predominantly occurred within the channel, which is indicated by a central peak in intensity. The spatial distribution broadened over time and more distant gel regions exhibited an increase in fluorescence intensity due to diffusion of the fluorescent protein into the gel. The profile plots show that the proteins diffuse over a mm length scale within a couple of hours, which indicates a diffusion coefficient on the order of *D* ≈ 10–100 μm^2^/s. For comparison, the diffusion coefficient of GFP in water had been previously determined as *D* ≈ 87 μm^2^/s,^51^ suggesting that protein diffusion was only slightly hindered in our gel. Notably, throughout the 16 h observation period, there was no equilibration in protein concentration: the interplay of continual protein synthesis in the center and diffusion into the gel resulted in a persistent concentration gradient, as also illustrated in the final heatmap in Figure 3b.

As a more complex system, we next investigated a gene circuit comprised of two plasmids with different spatial distribution (Figure 3e–h and SI Figure S8d–f). A plasmid expressing the fluorescent reporter mTur under the control of a σ_28_-specific *E. coli* promoter was mixed with the hydrogel. The other plasmid, encoding the corresponding σ_28_ under the control of a pTet promoter, was injected into the central channel. In the absence of TetR repressor, σ_28_ is expressed constitutively within the channel. Production of the mTur reporter only occurs when σ_28_ becomes available for binding to its promoter region, activating transcription.

We analyzed the spatial gene expression pattern as before. The resulting profile plots (Figure 3h and SI Figure S8e,f) show that reporter protein was produced throughout the gel also when the two circuit components were spatially separated. Similar as the mSc protein in the previous set of experiments, σ_28_ expressed in the central channel can diffuse into the agarose gel and activate transcription of the reporter protein there. Both the profile plots and the final heat map (Figure 3f) suggest a more evenly distributed increase in fluorescence intensity than observed for the mSc expression shown in Figure 3d. The profile plots further show a delayed expression of mTur, which is expected for such a cascaded circuit.

We also note that mTur fluorescence intensity is maximal in the center of the gel, i.e., in the region of the supply channel. Fluorescent protein produced inside the gel can also enter the central channel. Due to fast diffusion and the comparatively small width of the channel, there is no distinct spatial structure emerging in the profile plot.

We finally investigated a negative feedback circuit comprised of four plasmids (Figure 3i–k, SI Figure S9a,b), which had been previously used to implement a cell-free genetic oscillator in a microfluidic setup.^30^ The core circuit is composed of σ_28_ as a transcriptional activator, which is produced from one of the plasmids under the control of the pTet promoter. Conversely, the repressor protein, TetR, is produced from a second plasmid and is regulated by the p28 promoter. For readout, two additional plasmids are used to express mTur from a σ_28_ promoter, and mVenus from a pTet promoter. All four plasmids were injected into the central channel, leading to expression of the proteins in the center (Figure 3j), followed by diffusion of the protein products into the surrounding hydrogel. In this case, the hydrogel merely acts as a drain for the proteins, and as a reservoir for the prolonged supply of the reaction in the center with components of the cell-free expression system, but has no role in gene expression itself. We therefore focused on the evolution of the fluorescence intensity in the channel area only. A plot of the expression kinetics of the two reporter proteins is shown in Figure 3k.

After a short initial rise of the intensity values for both reporter proteins, mTur expression decreases until about 4 h after the start of the experiments, and then steeply rises almost linearly for the rest of the experiment. By contrast, the mVenus signal continuously rises over the course of >20 h, but the expression rate appears to decelerate as soon as mTur production ramps up. The initial dynamics are reminiscent of the initial phase of a genetic oscillator, which, however, cannot fully unfold due to the limited lifetime of the cell-free reaction in the system. Importantly, a control experiment, in which the plasmid coding for TetR is omitted, results in a “conventional” gene expression behavior, in which both mTur and mVen signals increase and saturate in synchrony (SI Figure S10).

### Nutrient Supply and Spatial Organization through Vascularization

One of the crucial design considerations for the generation of spatially extended, biochemically active materials is their efficient supply with nutrients and removal of waste products. Depending on the length scale, different transport mechanisms can be utilized: diffusion is effective only at short length scales, while for larger lengths active transport using molecular motors or fluid flow have to be employed.

In nature, branched fluid distribution networks are frequently utilized to supply large tissues homogeneously with chemicals or to target specific regions. Inspired by such networks, we were interested in generating spatially structured or homogeneous gene expression activity in a gel by employing asymmetric and branched structures rather than a central supply channel as in the previous experiments. To fabricate such networks, we utilized the indirect printing technique described in Figure 1.

**Figure 4:**
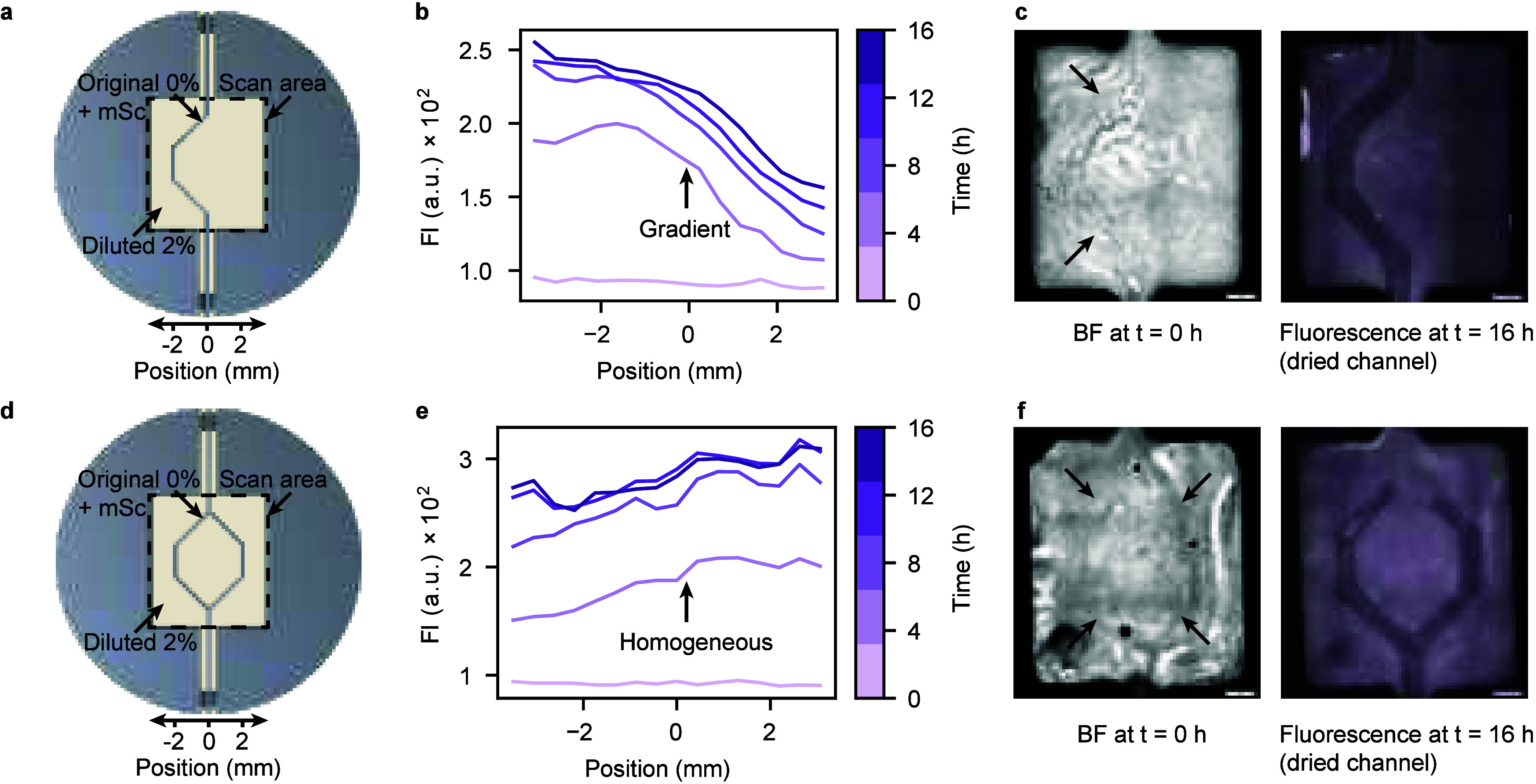
Protein synthesis in printed channel structures. The channels were filled with original mixtures comprising 75% cell-free extract and buffer plus mScarlet-I (mSc) template plasmid. The hydrogels consisted of the diluted mixture with 2% agarose and 37.5% extract and buffer. (a–c) Single-channel design with fluorescence intensity profile plots over time and microscopy images. To better visualize the channel structure and document their stability during the experiment, the fluorescence image was taken subsequent to the profile plot measurement, after the channels were dried. (d–f) Design with branching dual-channel, corresponding fluorescence intensity profile plots over time, and microscopy images. The profile plots were generated by averaging along the vertical orientation of the scan areas and plotting versus the horizontal coordinates. Arrows in the brightfield (BF) images indicate the boundaries of hydrogel channels filled with the cell-free reaction mixture.

We created a branched structure by splitting the central supply channel close to the entry into the gel into two arms and merged them again close to the exit (Figure 4). We compared gene expression in the gel in a situation where only one of the two arms, supplying the left half of the gel, was present (Figure 4a–c, SI Figure S10a–c), and using a setup in which both arms were used (Figure 4d–f, SI Figure S10d–f). As before, we supplied cell extract (original mixture with 0% agarose) together with plasmid containing a gene coding for mSc via the vasculature, while the gel itself contained diluted mixture with 2% agarose. We then monitored the resulting mSc expression profiles over 16 h to visualize the spatial distribution of the protein products.

As expected, for the single-arm structure clear variations in protein concentration were observed across the gel, with an almost constant level in the left half of the chamber, and an approximately linear concentration gradient generated by diffusion into the right half (Figure 4b). Conversely, the branched two-arm structure caused a comparatively uniform protein distribution throughout the whole gel area (Figure 4e). The final fluorescence intensity levels were notably lower compared to the cast channels presented in Figure 3. Considering the protein synthesis yield in the presence of Pluronic F-127 (SI Figure S3), we expect leftover sacrificial structures to impact gene expression to a lower degree. We therefore hypothesize that removal of the sacrificial Pluronic during fabrication might have also removed a fraction of the cell-free reaction mixture from the agarose hydrogel.

We wish to point out that the establishment of a stable protein gradient with long-term constant slope as in Figure 4b (and SI Figure 10b,c) requires the interplay of production in the channel and diffusion through the gel. Such a concentration profile could be used, e.g., as a synthetic morphogen gradient to control downstream differentiation events in the gel material, similar as in early embryonic development. Notably, in the context of Drosophila development, the long-term stability of such a gradient has been pointed out to be crucial for the precision of spatial positioning.^52^

## Conclusions

Fluidic networks are a common feature of complex multicellular organisms as they allow the supply and distribution of oxygen, nutrients, and signal molecules like hormones over large distances. Incorporating such structures into synthetic biomaterials represents a key challenge for the creation of spatially extended active systems with biological functionality. Utilizing cell-free gene expression in a spatial context offers the possibility to produce proteins *in situ*, upon demand and at different locations, potentially following an embedded gene regulatory program. However, such an approach poses significant challenges in terms of resource supply and usage, in particular with respect to the large reagent volumes required.

In this work, we integrated a cell-free transcription-translation system into bioprinted vascular hydrogels. We demonstrated the production of fluorescent reporter proteins in the gels, as well as the functionality of synthetic gene circuits based on both activation and repression. The dilution of cell extract upon gel addition as well as the final gel density were found to be important factors influencing the protein expression performance. Our homemade *E. coli*-based cell extract was functional within agarose using both standard and diluted concentrations (Figure 2). We found that the porous gel matrix only slightly impaired protein synthesis in our standard reaction mixture. The diluted reactions showed an overall reduced protein yield but were enhanced in the presence of the gel matrix. Our findings provide valuable insights into the conditions required for integrating cell-free expression within hydrogels, enabling us to fabricate vascular hydrogels that locally synthesize proteins and establish long-term protein concentration gradients without active flow (Figure 3). We successfully integrated an indirect printing approach (Figure 4) as well as synthetic gene circuitry, which demonstrates the potential of our approach to achieve designed self-organizing pattern formation in the future.

Although fabricating hydrogel networks on the cm-scale could be readily accomplished with the printing technique described (Figure 1), we restricted the spatial extension of our networks to a few millimeters to minimize the consumption of valuable cell-free components. For that purpose, we further utilized a gel matrix containing a diluted cell-free reaction mixture. Despite its reduced protein yield, it enabled operation of our systems for extended periods of time. On the long run, the cost-effective integration of large cell-free reaction volumes into bioprinted structures will necessitate larger scale production of cell extract and buffer components, which could be realized using lab automation in dedicated biofoundries.^20^ Employing a more highly concentrated cell-free expression system, such as by dissolving dried extracts, would further enable the higher protein yields necessary to supply more extended tissue-like structures.

We found the integration of synthetic genetic programs a promising route, that could allow spatiotemporal (4D) control of the resulting materials in the future. This could be used to realize environmentally responsive, shape-morphing hydrogel objects,^53^ which could autonomously sense, generate and release proteins or other molecules. The cell-free expression of enzymes that interact with the gel matrix as well as the compatibility of functional molecules within the hydrogel therefore needs to be assessed. Perfusion through the channel structures would sustain such reactions over extended periods of time, as demonstrated before in PDMS-based microfluidic systems.^29,30^ This would allow the realization of long-lived synthetic tissues and potentially interfacing of living with synthetic biological materials.^54^

## Methods

### Preparation of Cell Extract

In our cell-free gene expression experiments, we used homemade crude *E. coli* cell extract, based on a BL21-Rosetta 2 (DE3) strain. This strain was previously pORTMAGE engineered to be LacI deficient to include pLac promoter based plasmids.^55^ The extract was prepared following a previously introduced protocol.^18,42^ In a nutshell, the bacteria were cultivated in a Minifors 2 (Infors HT) 2 L bioreactor with *pO*_2_ and pH control. Cells were harvested at OD 6.0, and the suspension was first incubated with lysozyme then sonicated to achieve effective cell lysis while avoiding excessive heating. Specifically, we used 0.8 mg mL^–1^ lysozyme and 16 sonication cycles, which was reported previously to yield the optimum result with 14 mg mL^–1^ protein and YFP expression of 22 μM from a constitutive promoter. The cell extract was purified by dialysis and stored at −80 °C. We used one batch for all measurements with an extract protein content of 12 mg mL^–1^, assessed in a BCA-assay (Microplate BCA Protein Assay Kit - Reducing Agent compatible, Pierce) quality control (see SI Figure S11a). The activity of the extract was ensured by measuring the fluorescence intensity increase upon mScarlet-I expression in a standard 0% original reaction (see SI Figure S11b). The final intensity corresponds to a protein expression yield of approx. 5 μM (0.13 mg mL^–1^).

### Cell-Free Gene Expression in Hydrogels

The genetic parts applied here have previously been employed as parts of induction-repression based gene circuits.^30,55^ Plasmids cloned into *E. coli* DH5α were miniprepped and purified by phenol-chloroform extraction. The final plasmid concentration was 2 nM in all cell-free reactions.

The standard reaction and original mixtures consisted of 33% cell extract and 42% buffer solution, with the latter providing optimal reaction conditions and supplying the essential building blocks for transcription and translation.^18,42^ The final reaction contained the following: 50 mM Hepes pH 8 (#H6147-25G), 1.5 mM ATP (Roth, #HN35.3) and GTP (Roth, #K056.4), 0.9 mM CTP (Roth, #K057.4) and UTP (Roth, #K055.3), 0.2 mg/mL tRNA (Merck, #10109541001), 0.26 mM coenzyme A (Merck, #C3144-10MG), 0.33 mM NAD+ (Merck, #481911), 0.75 mM cAMP (Merck, #A9501-1G), 68 μM folinic acid (Merck, #47612-250MG), 1 mM spermidine (Merck, #S2626-1G), and 30 mM 3-PGA (Merck, #P8877-1G), as an energy source. Four mM Mg-glutamate (Merck, #49605-250G), 60 mM K-glutamate (Merck, #49601-100G), and 0 mM DTT, 1.5 mM of each amino acid except leucine and 1.25 mM leucine (Biozym, #BR1401801), 2.5% (w/v) PEG-8000 (Merck, #89510-250G-F).

In the diluted mixtures, we added the same volume of nf water or agarose so that all concentrations were reduced by half to 16.5% cell extract and 21% buffer solution. All reactions were measured over time at *T* = 29 °C.

### Hydrogel Mixtures

We utilized Agarose Super LM ROTIGarose (Roth #HP45.1) as gel matrix in all agarose samples. Stocks of varying concentrations (2%, 4%, 8%) were prepared by dissolving the powder in nf water and heating to 80 °C. For cell-free gene expression experiments, the agarose was cooled to 37 °C to prevent protein denaturation while mixing later on. In parallel, the cell-free reaction components were prepared on ice with 33% extract and 42% buffer. To prevent an immediate gelation of agarose while blending, the cell-free reaction was heated to 37 °C beforehand. The reaction mix was then added to the agarose at 37 °C by using a piston pipet and mixed by pipetting up and down ten times. For the original mixtures, the agarose was diluted four times during that step, for the diluted mixtures two times to achieve final agarose concentrations of 1% or 2%. The mixtures were transferred to either a 384-well plate (in expression characterization experiments) or to custom-printed PLA-chambers (spatiotemporal expression experiments). Finally, the constructs were cooled to 4 °C for 10 min to ensure homogeneous gelation.

### Hydrogel Vascularization

Channels within agarose hydrogels were fabricated either by molding or by employing Pluronic F-127 (Sigma, #P2443-250G) as a fugitive ink. For straight channels, Sterican needles (Roth #X129.1) served as a mold. They were fixed in place within PLA chambers printed with an Ultimaker 3 Extended. Liquid agarose was cast on top and gelified at 4 °C so that the needle could be removed again, leaving behind a straight channel of approximately the same width and height. For the fabrication of more complex structures, we used a BioX (Cellink) 3D-bioprinter to print 40% Pluronic F-127. The ink was extruded at room temperature (RT) through a polypropylene conical nozzles of type 27G (Cellink, #NZ3270005001) corresponding to an inner diameter of 200 μm. This setup was used to generate filaments with a width ranging from 0.2 mm to 1 mm and corresponding height of 0.1 mm to 0.5 mm (when assuming a cylindrical filament with a circular cross section or a wetted filament with half an ellipse as a cross section). We printed onto glass slides or glass slips with the following typical printing parameters: 2 to 3 mm s^–1^, 100 ms preflow and 0 ms postflow, pressures around 150 kPa. The precise printing parameters for creating consistent structures varied depending on cartridge filling and structure design. PLA chambers printed with the Ultimaker 3 Extended were glued around the structure as containers, and liquid agarose was cast on top. The constructs were cooled to 4 °C to both solidify the agarose hydrogel and liquefy Pluronic F-127. We removed the sacrificial ink subsequently and injected the filling solution with fine dosage syringes Omnican-F (VWR, #720-2560). The designs for the sacrificial Pluronic and the PLA framing chambers were created in Inventor (Autodesk) and converted to .stl or .gcode files for printing.

### Fluorescence Scans, Profile Plots, and Data Analysis

Time-resolved changes of fluorescence were measured in a BMG Labtech CLARIOstar Plus fluorescence platereader. For cell-free performance assessment, the samples were measured in a 384-well plate. The data were processed and evaluated with Python standard libraries. For the curve fit, we used SciPy optimize. A detailed description of the fitting assumptions and the kinetic model can be found in the Supporting Information.

Probes with vascularized hydrogels were fabricated on 20 mm and placed into 12-well plates for scanning using the same platereader. A circular scan area with a diameter of 12 mm was covered, having a resolution of 30 × 30 data points. The data was cut to the actual scan area in Python, and profile plots were created by averaging the vertical values.

### Microscopy

We imaged the large fluorescein-filled networks with an EVOS M7000 microscope in the GFP channel at 4× magnification. A grid of 9 × 6 tiles was scanned with a 10% overlap, and images were processed in Fiji. For shading correction, we employed the BaSiC tool,^56^ and the grid/collection stitching plugin was used to assemble the tiles.

Smaller structures incorporating cell-free gene expression were imaged with a Nikon Ti-2E fluorescence microscope, equipped with a SOLA SM II LED light source, an Andor NEO 5.5 camera, and controlled with NIS-Elements software. Using a 4× magnification objective, we captured 3 × 3 tiles and applied the internal shading correction and stitching of the software.

## Supporting Information Available

Additional data sets and replicates of experiments (Figures S1–S3 and S8–S10), a description of the applied kinetic modeling, data fits for parameter determination (Figures S4–S6), setup images (Figure S7), and a quality control of the cell extract used (Figure S11) (PDF)
