# Supplementary material for: Cell-Free Gene Expression in Bioprinted Fluidic Networks
Source: ACS Synth Biol. 2024 Jul 23;13(8):2447–56. doi: 10.1021/acssynbio.4c00187 (PMC11334185; doi:10.1021/acssynbio.4c00187)
Supplement: Supplementary file 1 — sb4c00187_si_001.pdf [file sb4c00187_si_001.pdf]

Cell-free Gene Expression in Bioprinted Fluidic Networks

Supporting Information

Alexandra Bienau, Anna C. Jäkel, and Friedrich C. Simmel*

TU Munich, School of Natural Sciences, Department of Bioscience, Garching, Germany

E-mail: simmel@tum.de

Phone: +49 89 289 11610. Fax: +49 89 289 11612

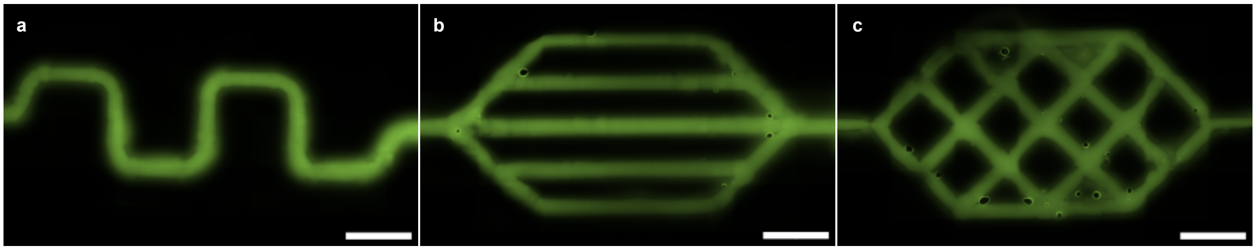

Figure S1: Fluorescein-filled channel structures in agarose hydrogel. We printed sacrificial structures consisting of Pluronic F-127 and cast preheated agarose on top. The Pluronic ink could be removed after cooling to $4°$, leaving vascularized hydrogels that were filled with fluorescein solution for visualization. Various designs of different complexity are shown here, ranging from a simple curved serpentine (a), a branched structure with parallel channels (b), and a grid network (c). All scale bars represent $3\,\mathrm{mm}$.

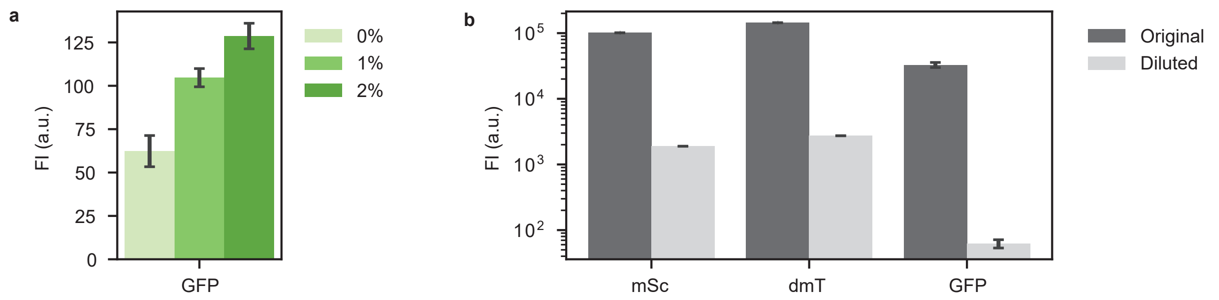

Figure S2: Commercial myTXTL cell extract in agarose hydrogels. (a) Fluorescence intensity values for cell-free protein synthesis of GFP diluted mixtures of myTXTL cell extract after $16\,\mathrm{h}$. We readily prepared reactions of the myTXTL Sigma 70 Master Mix together with the provided control plasmid P70a-deGFP and added the same volume nf water (0% sample) or agarose (1% and 2% sample) to create diluted mixtures with the given final agarose concentrations. (b) Comparison of final fluorescence intensity values between the diluted and original 0% samples. The mScarlet-I (mSc) and mTurquoise2 (mTur) proteins were produced with our housemade *E. coli* based cell extract as described before. GFP was synthesized in commercial myTXTL Sigma 70 Master Mix with the control plasmid P70a-deGFP. The original samples were prepared with standard concentrations. For the diluted samples, the concentrations of all components except the template plasmid were halved by adding the same volume nf water.

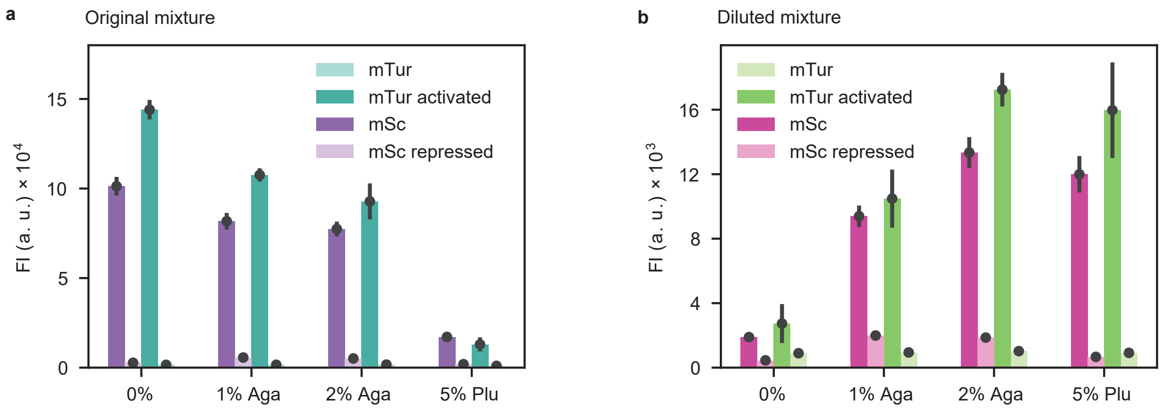

Figure S3: Gene expression activation and repression. A plasmid expressing the fluorescent reporter mScarlet-I (mSc) under the control of a Tet promoter was measured with and without an additional plasmid encoding the TetR protein, repressing mSc production. Another plasmid with the reporter mTurquoise2 (mTur) controlled by a σ_{28}-specific promoter was measured with and without an additional plasmid encoding the sigma factor 28 (σ_{28}). Expression of mTur is active when this factor is present. (a) Fluorescence intensity values for cell-free protein synthesis after $16\,\mathrm{h}$ in original mixtures with standard concentrations. (b) Final fluorescente intensity values in diluted mixtures. The sample were measured in a 0% control, 1% and 2% agarose gels, and 5% Pluronics F-127. The control was created by adding the same volume nf water, the others by diluting with corresponding gel stocks.

Kinetic modeling

We consider a simple kinetic model for the expression of fluorescent proteins in the cell extract that accounts for the degradation of resources over time. We will use the following abbreviations for the involved species and their concentrations. $[\mathrm{R}]$ denotes the concentration of RNA, $[\mathrm{D}]$ the constant concentration of plasmid DNA provided ($2\,\mathrm{nM}$), $[\mathrm{P}]$ is the concentration of the nascent fluorescent protein P, while $[\mathrm{F}]$ is the concentration of the fluorescent protein F after the maturation step. The recorded signals are proportional to $[\mathrm{F}]$. We further introduce the dimensionless variable X that accounts for the availability of resources in the extract.

Transcription, translation and protein maturation are modeled by the following equations:

$$\frac{d[\mathrm{R}]}{dt} = \alpha_R[\mathrm{D}] - \delta_R[\mathrm{R}] \quad (1)$$

$$\frac{d[\mathrm{P}]}{dt} = \alpha_P[\mathrm{R}] \cdot X - \delta_1[\mathrm{P}] \quad (2)$$

$$\frac{d[\mathrm{F}]}{dt} = \delta_1[\mathrm{P}] \quad (3)$$

Here, α_R, α_P are the RNA and protein production rates, δ_R is the RNA degradation rate. The term δ_1 corresponds to the maturation rate of the fluorescent protein. Moreover, protein production (Eq. 2) is assumed to depend on the presence of RNA and the availability of resources, which are measured by the (dimensionless) parameter X. As mentioned in the main text, our experiments indicate that the fluorescent proteins are stable on the time scale of the experiment, and we thus omit a degradation term. We assume simple first order degradation of the resource term:

$$\frac{dX}{dt} = -\delta_2 X, \quad (4)$$

which leads to

$$X(t) = X_0 e^{-\delta_2 t}, \quad (5)$$

The first equation has the solution:

$$[\mathrm{R}](t) = \frac{\alpha_R}{\delta_R} \cdot (1 - e^{-\delta_R t}). \tag{6}$$

As RNA is expected to be unstable in the cell extract ($\delta_R > \delta_1, \delta_2$), we make the simplifying assumption that RNA is present at its steady state concentration $[\mathrm{R}]_{ss} = \alpha_R/\delta_R$.

This reduces our initial model (Eq. 1-4) to:

$$\frac{d[\mathrm{P}]}{dt} = \alpha \cdot e^{-\delta_2 t} - \delta_1[\mathrm{P}] \tag{7}$$

$$\frac{d[\mathrm{F}]}{dt} = \delta_1[\mathrm{P}], \tag{8}$$

where $\alpha := \alpha_R \alpha_P X_0/\delta_R$. α has dimensions of $1/\mathrm{h}$.

The first equation has the solution (initial condition $[\mathrm{P}](0) = 0$):

$$[\mathrm{P}](t) = \frac{\alpha}{\delta_1 - \delta_2}\left(e^{-\delta_2 t} - e^{-\delta_1 t}\right) \tag{9}$$

Integrating of Eq. 8 then gives, with $[\mathrm{F}](0) = 0$,

$$[\mathrm{F}](t) = \frac{\alpha}{\delta_2} \cdot \frac{\delta_1(1 - e^{-\delta_2 t}) - \delta_2(1 - e^{-\delta_1 t})}{(\delta_1 - \delta_2)} \tag{10}$$

which is the Equation (1) used in the main text.

For further analysis, we normalized the data by dividing through the end level value ξ, which is:

$$\xi = \lim_{t\to\infty}[\mathrm{F}](t) = \frac{\alpha}{\delta_2} = \frac{\alpha_R \alpha_P X_0}{\delta_R \delta_2} \tag{11}$$

This endpoint normalization yields an expression $[\mathrm{F}](t)/\xi$, which reduces the number of fitting parameters from three to two (δ_1 and δ_2) to avoid over-fitting. In Figure S4-S6, we show fits of that expression to the data for different values of δ_1 and δ_2, for the original and diluted samples for different agarose concentrations.

Remarks

- The fits show overall good agreement with the data, with realistic values for the lifetime of the cell extract (on the order of 4-5 hours in the 0% samples, and 7-10 hours in the gel containing samples). With about 40-50 min, the time scale for the second fit parameter δ_1 corresponds well with that expected for the maturation of a fluorescent protein.

- It is impossible to fit the sigmoidal shape of the fluorescence time courses with a single rate parameter. We also considered other processes that could generate the initially observed lag phase. In fact, there is no clear time scale separation between the RNA degradation time scale $(1/\delta_R)$, which is expected to be on the order of minutes up to tens of minutes, and the time required for protein maturation $(1/\delta_1)$. Omitting the protein maturation step, and instead including RNA degradation, results in the following, alternative expression for the fit function:

$$[\mathrm{F}](t) = \xi \times \left[1 - e^{-\delta_2 t}\left(1 + \frac{\delta_2}{\delta_R}(1 - e^{-\delta_R t})\right)\right]$$

  Fitting this function appeared to result in unrealistically long RNA lifetimes on the order of hours, however.

- One could include both processes - RNA degradation and protein maturation - in the fitting procedure, but many combinations of δ_1 and δ_R would result in similarly good fits. To avoid overfitting, we decided to only use two fit parameters, bearing in mind that the fitted parameter might not only represent the maturation phase of the fluorescent protein, but contain also contributions by the RNA transcription and degradation process.

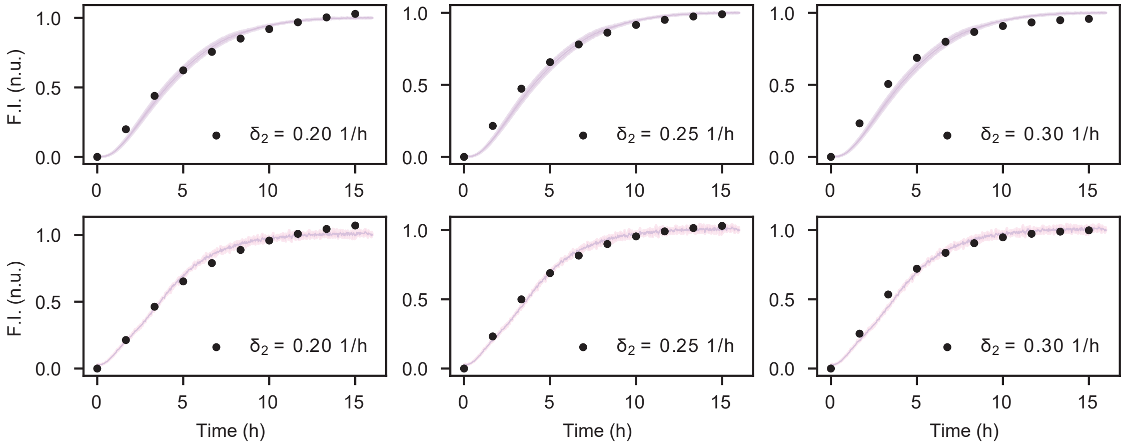

Figure S4: Comparing different δ_2 values in the normalized kinetic fit of 1% samples. The normalized protein production was fitted to the kinetic data of mScarlet-I (mSc) gene expression with fixed guess-values for the parameter δ_2. A range of 0.2 - 0.3 1/h was compared for the original 0% sample set (upper plots), and 0.2 - 0.3 1/h for the diluted 0% sample set.

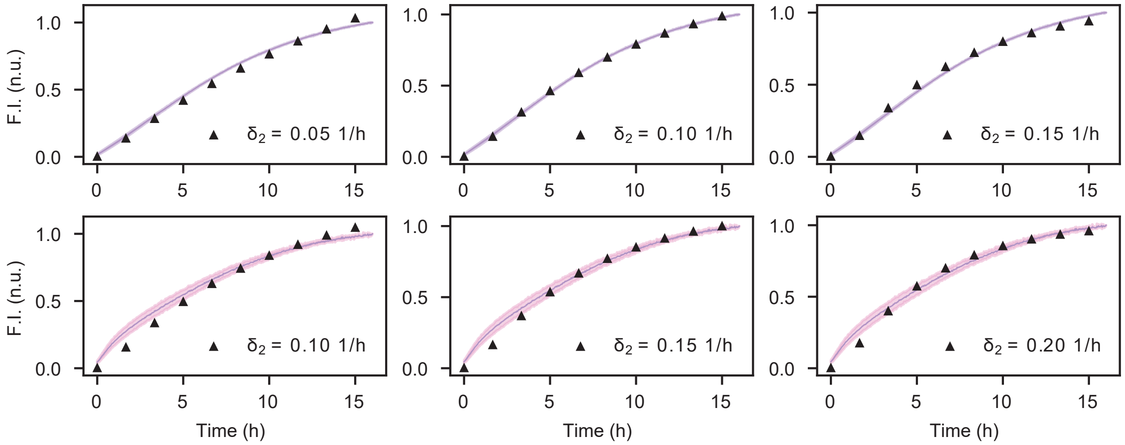

Figure S5: Comparing different δ_2 values in the normalized kinetic fit of 2% samples. The normalized protein production was fitted to the kinetic data of mScarlet-I (mSc) gene expression with fixed guess-values for the parameter δ_2. A range of 0.05 - 0.15 1/h was compared for the original 1% sample set (upper plots), and 0.1 - 0.2 1/h for the diluted 1% sample set.

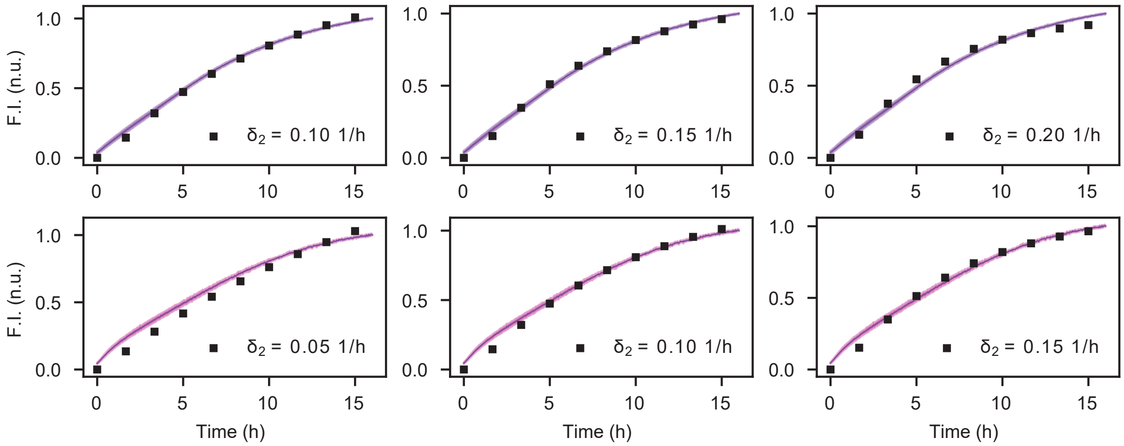

Figure S6: Comparing different δ_2 values in the normalized kinetic fit of 0% samples. The normalized protein production was fitted to the kinetic data of mScarlet-I (mSc) gene expression with fixed guess-values for the parameter δ_2. A range of 0.1 - 0.2 1/h was compared for the original 2% sample set (upper plots), and 0.05 - 0.15 1/h for the diluted 2% sample set.

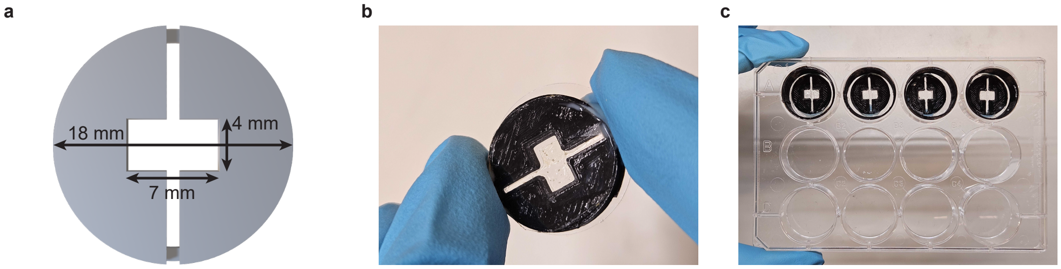

Figure S7: Single-channel chamber. (a) Illustration of the container design with dimensions. (b) Photograph of single sample. The container was glued to a circular glass slip with diameter $20\,\mathrm{mm}$ and filled with agarose. The chamber surface was sealed with oxygen permeable foil and the inlets with Picodent. (c) Photograph of four samples placed into a 12-well plate. A cylindrical PLA cover was applied to each relevant well for avoiding sample-crosstalk.

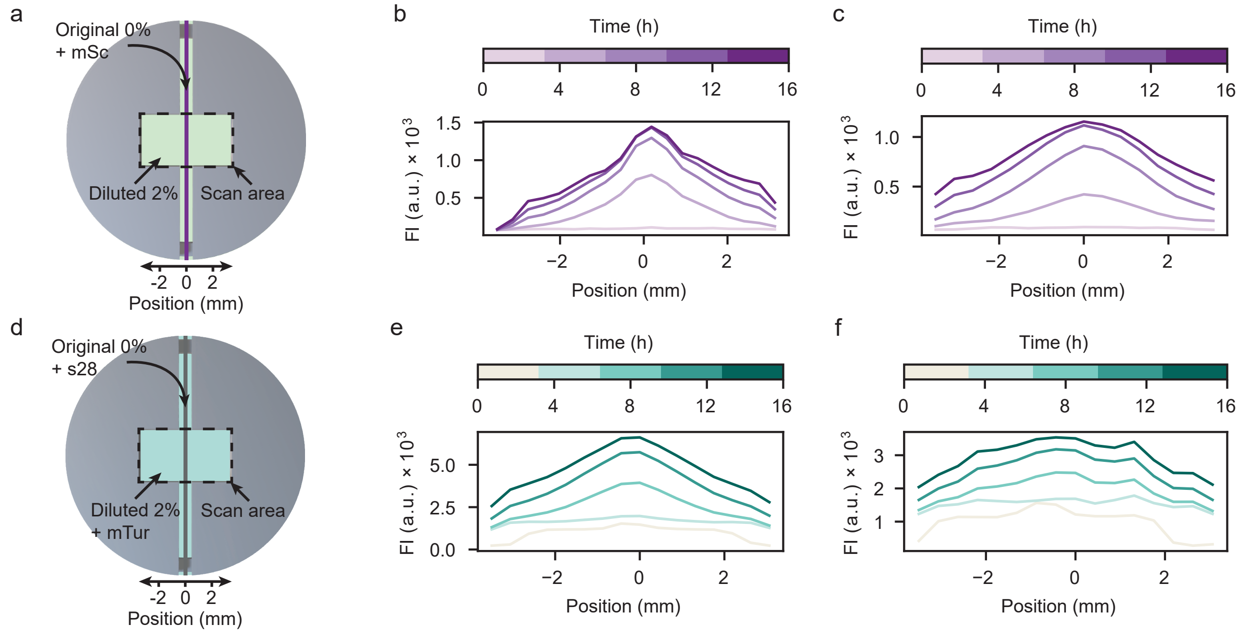

Figure S8: Additional datasets to Figure 3 of cell-free protein synthesis and diffusion in single-channel hydrogels. The samples were constructed as shown in the schematics (a,d) featuring a central channel within an agarose hydrogel. These channels were filled with original mixtures containing 75 % cell-free extract and buffer plus the indicated plasmids. The hydrogels were composed of the diluted mixture with 2 % agarose, 37.5 % extract and buffer, and for (d) additionally of mTurquoise2 (mTur) plasmid. (b-c) Profile plots over time of mScarlet-I (mSc) fluorescence intensity, obtained by averaging the scan area over the vertical direction. (e-f) Temporal profile plots showing mTurfluorescence intensity averaged over the vertical direction. Gene expression of the reporter was activated by the σ_{28} produced in the channel only.

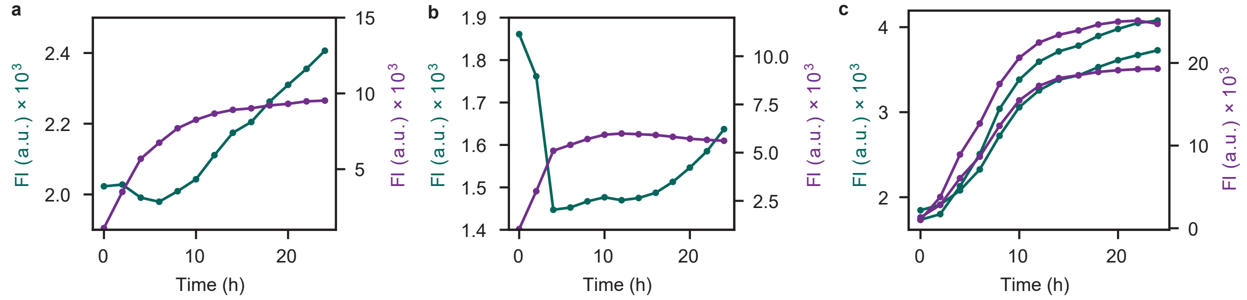

Figure S9: Additional datasets to Figure 3 and control of feedback gene circuit. (a-b) Plasmids encoding the two regulatoray proteins sigma factor (σ_{28}) and repressor TetR, and two reporter proteins mTurquoise2 (mTur) and mVenus (mVen) were supplied into central channels of agarose hydrogel supplemented with cell-free reaction mixture. The fluorescence intensity within the channel area was monitored and the average value plotted over time. (c) Two control datasets without the plasmid encoding the repressor TetR. The other three plasmids of the circuit were injected within cell-free reaction mixture into agarose hydrogel channels.

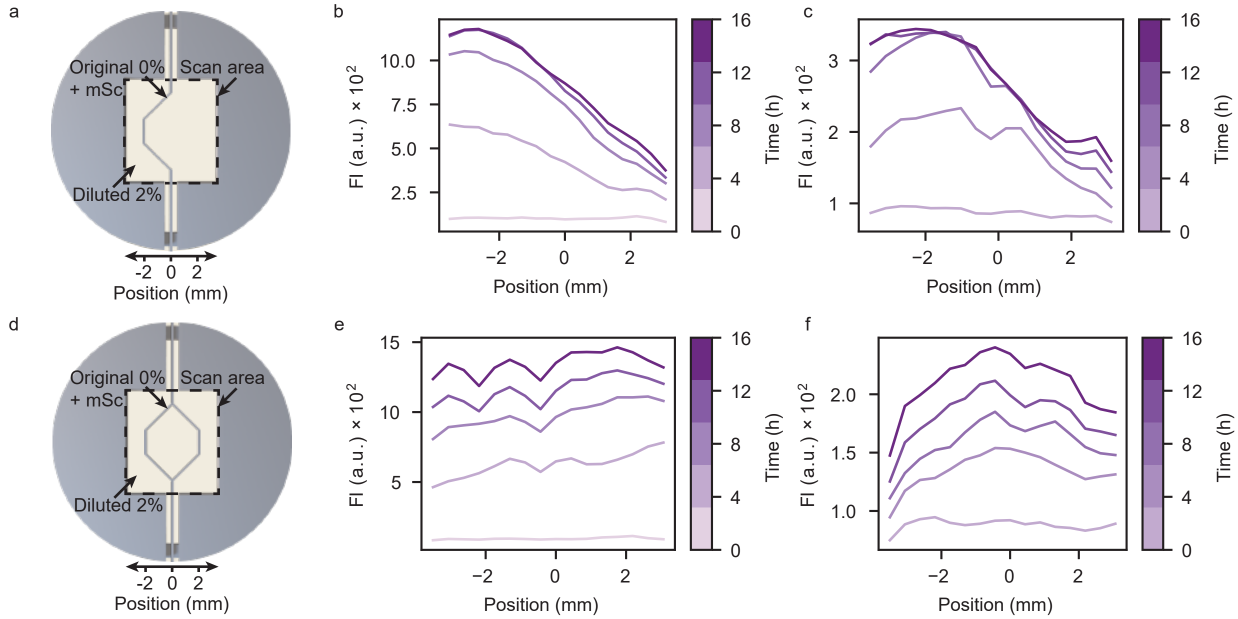

Figure S10: Additional datasets of protein synthesis in printed channel structures. The channels were filled with original mixtures comprising 75 % cell-free extract and buffer plus mScarlet-I (mSc) template plasmid. The hydrogels consisted of the diluted mixture with 2 % agarose and 37.5 % extract and buffer. (a-c) Single-channel design with temporal fluorescence intensity profile plots, generated by averaging the scan area over the vertical direction. (d-f) Dual-channel design and corresponding fluorescence intensity profile plots over time, created by vertical averaging.

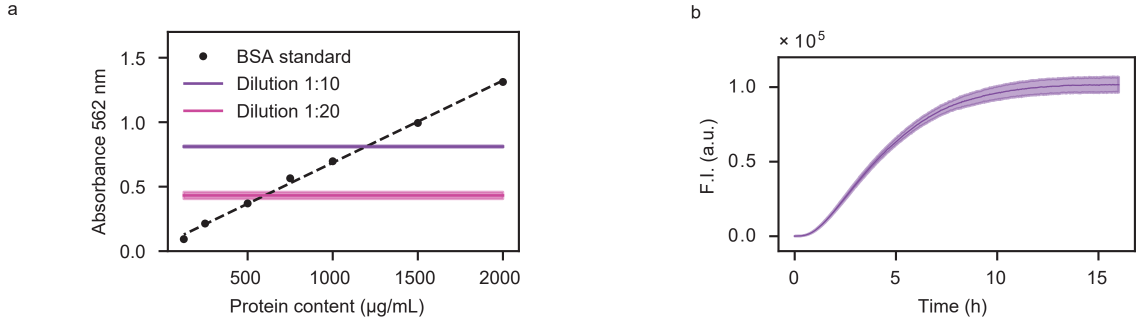

Figure S11: Cell extract quality control. (a) We measured the protein content of our 1:10 and 1:20 diluted homemade cell extract in a BCA-assay using a bovine serum albumin (BSA) standard. (b) The extract activity was evaluated by measuring the increase in fluorescence intensity by mScarlet-I (mSc) protein expression within the 0 % original sample.
